# Consensus statement on the definition of neurogenic supine hypertension in cardiovascular autonomic failure by the American Autonomic Society (AAS) and the European Federation of Autonomic Societies (EFAS): Endorsed by the European Academy of Neurology (EAN) and the European Society of Hypertension (ESH)

**DOI:** 10.1007/s10286-018-0529-8

**Published:** 2018-05-15

**Authors:** Alessandra Fanciulli, Jens Jordan, Italo Biaggioni, Giovanna Calandra–Buonaura, William P. Cheshire, Pietro Cortelli, Sabine Eschlboeck, Guido Grassi, Max J. Hilz, Horacio Kaufmann, Heinz Lahrmann, Giuseppe Mancia, Gert Mayer, Lucy Norcliffe–Kaufmann, Anne Pavy–Le Traon, Satish R. Raj, David Robertson, Isabel Rocha, Walter Struhal, Roland Thijs, Konstantinos P. Tsioufis, J. Gert van Dijk, Gregor K. Wenning

**Affiliations:** 1 Department of Neurology, Innsbruck Medical University, Anichstraße 35, 6020 Innsbruck, Austria; 2 German Aerospace Center and Chair of Aerospace Medicine, Institute of Aerospace Medicine, University of Cologne, Cologne, Germany; 3 Division of Clinical Pharmacology, Vanderbilt University, Nashville, USA; 4 Department of Biomedical and Neuromotor Sciences, University of Bologna, Bologna, Italy; 5 IRCCS, Institute of Neurological Sciences of Bologna, Bologna, Italy; 6 Department of Neurology, Mayo Clinic, Jacksonville, USA; 7 Clinica Medica, University of Milan-Bicocca, Milan, Italy; 8 Istituto di Ricerca a Carattere Scientifico IRCCS Multimedica, Sesto San Giovanni, Milan Italy; 9 Department of Neurology, Universitätsklinikum Erlangen, Erlangen, Germany; 10 Department of Neurology, Icahn School of Medicine at Mount Sinai, New York, USA; 11 Department of Neurology, Dysautonomia Center, New York University School of Medicine, New York, USA; 12 Private Practice, Vienna, Austria; 13 Centro di Fisiologia Clinica ed Ipertensione, Milan, Italy; 14 Department of Internal Medicine IV, Innsbruck Medical University, Innsbruck, Austria; 15 Department of Neurology, French Reference Centre for Multiple System Atrophy, University Hospital of Toulouse, Toulouse, France; 16 UMR INSERM 1048, Toulouse, France; 17 Department of Cardiac Sciences, Libin Cardiovascular Institute of Alberta, University of Calgary, Calgary, Canada; 18 Institute of Physiology, Faculty of Medicine, University of Lisbon, Lisbon, Portugal; 19 Department of Neurology, Karl Landsteiner University of Health Sciences, Site Tulln, Tulln, Austria; 20 Stichting Epilepsie Instellingen Nederland, Heemstede, The Netherlands; 21 Department of Neurology, Leiden University Medical Centre, Leiden, The Netherlands; 22 1st Department of Cardiology, Hippokration General Hospital, National and Kapodistrian University of Athens, Athens, Greece

**Keywords:** Neurogenic supine hypertension, Nocturnal hypertension, Neurogenic orthostatic hypotension, ABPM, Autonomic failure

## Abstract

**Purpose:**

Patients suffering from cardiovascular autonomic failure often develop neurogenic supine hypertension (nSH), i.e., high blood pressure (BP) in the supine position, which falls in the upright position owing to impaired autonomic regulation. A committee was formed to reach consensus among experts on the definition and diagnosis of nSH in the context of cardiovascular autonomic failure.

**Methods:**

As a first and preparatory step, a systematic search of PubMed-indexed literature on nSH up to January 2017 was performed. Available evidence derived from this search was discussed in a consensus expert round table meeting in Innsbruck on February 16, 2017. Statements originating from this meeting were further discussed by representatives of the American Autonomic Society and the European Federation of Autonomic Societies and are summarized in the document presented here. The final version received the endorsement of the European Academy of Neurology and the European Society of Hypertension.

**Results:**

In patients with neurogenic orthostatic hypotension, nSH is defined as systolic BP ≥ 140 mmHg and/or diastolic BP ≥ 90 mmHg, measured after at least 5 min of rest in the supine position. Three severity degrees are recommended: mild, moderate and severe. nSH may also be present during nocturnal sleep, with reduced-dipping, non-dipping or rising nocturnal BP profiles with respect to mean daytime BP values. Home BP monitoring and 24-h-ambulatory BP monitoring provide relevant information for a customized clinical management.

**Conclusions:**

The establishment of expert-based criteria to define nSH should standardize diagnosis and allow a better understanding of its epidemiology, prognosis and, ultimately, treatment.

## Introduction

Primary cardiovascular autonomic failure develops in the context of inherited and sporadic neurodegenerative diseases affecting the autonomic nervous system. Secondary causes of cardiovascular autonomic failure include amyloidosis and metabolic or immune-mediated diseases inducing autonomic neuropathy [1].

The main feature of cardiovascular autonomic failure is neurogenic orthostatic hypotension (nOH), defined in 2011 as a sustained reduction of systolic blood pressure (BP) of ≥ 20 mmHg (≥ 30 mmHg in patients with supine hypertension) or diastolic BP  of ≥  10 mmHg within 3 min of standing or head-up tilt of at least 60° [2]. About one-half of the patients with nOH develop neurogenic supine hypertension (nSH), which can be severe and last several hours during sleep [3]. nSH is distinct from essential hypertension, since most patients with nSH are normotensive while seated and may be severely hypotensive while standing [4–6].

As yet, no consensus criteria have been established for the diagnosis of nSH, limiting current understanding of its epidemiology, prognostic significance and the development of appropriate management strategies.

To address this shortcoming, a round table discussion with participants from the European Federation of Autonomic Societies (EFAS) and from the American Autonomic Society (AAS) was convened in Innsbruck on February 16, 2017 to establish clinical criteria for the diagnosis of nSH in the context of cardiovascular autonomic failure.

Discussion points and statements originating from this meeting were subsequently examined and reviewed by representatives of both societies and are summarized in the document presented here. The final version was endorsed by the European Academy of Neurology (EAN) and by the European Society of Hypertension (ESH).

## Definitions

### Supine hypertension

In patients with proven nOH, nSH is defined as systolic BP of ≥ 140 mmHg and/or diastolic BP of ≥ 90 mmHg, measured after at least 5 min of rest in the supine position.

We propose the following ranges to define the severity of nSH in autonomic failure:

- Mild nSH: systolic BP values of 140–159 mmHg or diastolic BP values of 90–99 mmHg.
- Moderate nSH: systolic BP values of 160–179 mmHg or diastolic BP values of 100–109 mmHg.
- Severe nSH: systolic BP values of ≥ 180 mmHg or diastolic BP values of   ≥ 110 mmHg.

### Nocturnal hypertension

Patients with cardiovascular autonomic failure frequently show nSH also during sleep, i.e. nocturnal hypertension, with loss of the physiological nocturnal BP fall at night of ≥ 10% while supine and asleep (dipping). Two main pathological nocturnal BP profiles are distinguished:

- Reduced-dipping: characterized by a mean nocturnal BP reduction of < 10% with respect to mean daytime BP values.
- Non-dipping or rising: when the mean BP does not decrease or even increases during the night with respect to daytime [7, 8].

Additional aspects are important for an appropriate interpretation of 24 h-ambulatory BP monitoring (24 h-ABPM) in patients with cardiovascular autonomic failure and are discussed in the section Diagnostic work-up.

## Clinical features

Neurogenic supine hypertension is mostly asymptomatic or induces only non-specific complaints, such as headache. The main, most clinically relevant and immediate consequence of nSH is an exacerbation of pressure natriuresis during sleep, causing nocturia, sleep disturbances, volume depletion overnight and worsening of nOH in the morning [9, 10].

**Fig. 1:**
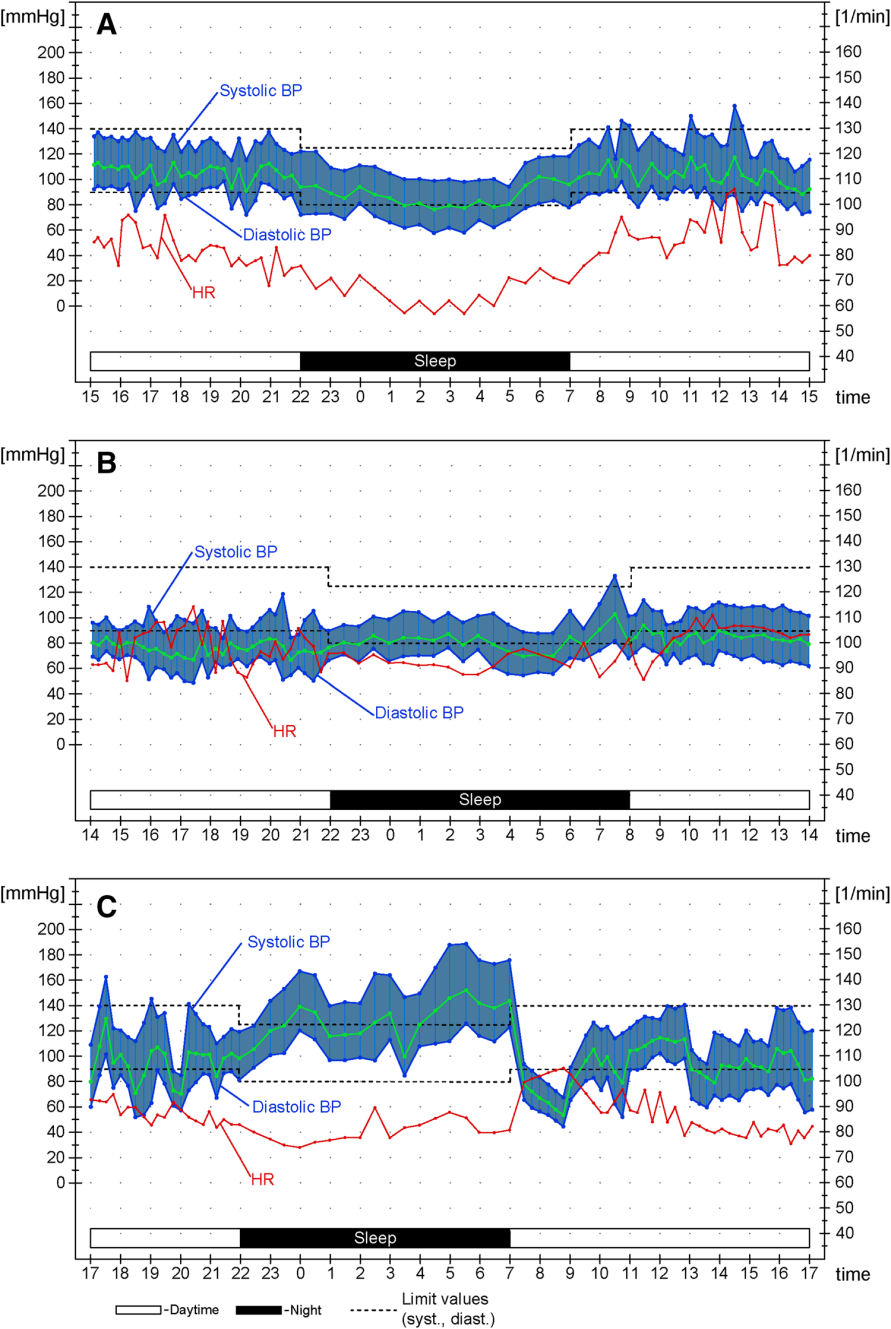
Nocturnal blood pressure (BP) profiles at 24 h-ambulatory BP monitoring **a** Physiological nocturnal dipping profile (mean nocturnal BP falls by ≥ 10% with respect to  daytime BP), **b** reduced-dipping profile (mean nocturnal BP falls by < 10% with respect to daytime BP), **c** rising profile (mean nocturnal BP increases with respect to daytime BP), in a patient with cardiovascular autonomic failure. Note severe hypotension occurring in the early morning.* HR* Heart rate. The blue area indicates the pulse pressure, the green line indicates the mean BP. Adapted from Fanciulli et al. [44], with permission of Springer SBM. This image is excluded from the creative commons license

nSH could potentially result in hypertensive emergencies, with similar complications as in the general population, i.e. cerebral hemorrhage, ischemic stroke, acute pulmonary edema and myocardial infarction [11], although the overall occurrence of these clinical events has not been systematically investigated in cardiovascular autonomic failure. Acute cardiovascular complications associated with nSH have been reported, but mainly in patients also receiving anti-hypotensive drugs [12, 13], which are known to induce or exacerbate nSH (Fig. 1).

The long-term effects of cardiovascular autonomic failure have been studied in patients with Parkinson’s disease (PD) and multiple system atrophy (MSA). Current evidence suggests that early-onset cardiovascular autonomic failure is associated with a poor prognosis [14–20], development of cardiovascular [21–23], kidney [24] and cerebrovascular [25–30] disease, as well as cognitive impairment [26, 27, 31–36]. Similarly, a higher prevalence of left ventricular hypertrophy, nephropathy and vascular and cerebrovascular disease has been reported in diabetic patients with secondary cardiovascular autonomic failure compared to those without [37].

At present, it is uncertain how much of the increase in risk is related to the underlying disease causing autonomic failure and how much is directly related to the abnormality in BP regulation. It is also unclear whether nOH or nSH represents the actual negative prognostic factor in patients with cardiovascular autonomic failure—or whether it is the BP volatility induced by the combination of both [38].

## Epidemiology

Keeping in mind the limitations due to inconsistences in the diagnostic criteria, nSH has been reported in 34–46% of patients with PD and 37% of patients with MSA [3, 39]. The frequency rates of nSH increase to 50% in parkinsonian patients with nOH, supporting the assumption of an etiological association between nOH and nSH [3, 39–42]. Loss of nocturnal BP dipping has been frequently reported in PD (48%) and MSA patients (up to 75%) [43–46].

The prevalence of nSH in pure autonomic failure (PAF) ranges from 48 to 70% [24, 47]. An abnormal, non-dipping, nocturnal BP profile has been described in up to 86% of patients with PAF [48].

To date, no studies have addressed the epidemiology of nSH in diabetes, but a significant association has been reported between the presence of nOH and rising nocturnal BP profiles both in patients with type 1 and type 2 diabetes mellitus [49, 50].

The prevalence of nSH in genetic and acquired amyloidosis, as well as in other kinds of autonomic neuropathies is unknown. Clinically, patients with acquired amyloidosis (AL) and nOH often present with relatively low supine BP measurements which may be related to cardiovascular amyloid deposition.

## Pathophysiology

Anti-hypotensive drugs used for the treatment of nOH may unmask or exacerbate nSH, thereby posing a management challenge. However, nSH can also develop in the absence of anti-hypotensive treatment, which reflects the likelihood that multiple factors contribute to nSH, including impairment of the afferent, central and efferent pathways of the arterial baroreflex arch, disruption of the renin–angiotensin–aldosterone axis and denervation supersensitivity at the level of the vascular adrenoceptors due to impaired sympathetic transmission [6, 41, 51–53].

Noradrenaline release from intact post-ganglionic sympathetic fibers in the setting of severe baroreflex impairment drives nSH in MSA, which is characterized in most cases by severe preganglionic sympathetic denervation, with sparing of the post-ganglionic sympathetic fibers to the heart and blood vessels [53].

In contrast, mechanisms independent of the sympathetic autonomic nervous system are considered to contribute to the development of nSH in autonomic disorders with post-ganglionic sympathetic denervation and very low circulating noradrenaline levels, such as in PAF [53]. Paradoxically increased angiotensin II and aldosterone receptor signaling in the setting of decreased activity of the systemic renin–angiotensin–aldosterone system has been implicated [51, 54]. In contrast, an impairment in nitric oxide-mediated vasodilation has not been observed in patients with nSH due to primary cardiovascular autonomic failure [55].

## Diagnostic work-up

All patients newly diagnosed with nOH should be screened for nSH at the time of diagnosis and at regular intervals afterwards, especially if they begin treatment with anti-hypotensive agents, increase their dosage, report multiple episodes of nocturia per night or develop ankle edema.

Office screening should be performed by measuring supine BP as soon as the patient assumes the supine position and then again after the patient has been resting supine for at least 5 min. This can be combined with a standing test, by having the patient stand immobile to avoid engaging the muscle pumps in the lower limbs, or with a tilt table examination. A tilt table examination may be preferred in patients unable to stand for several minutes.

It should be noted that the criteria outlined here to define nSH only apply for patients with proven nOH: if present, both conditions are usually verified during the same test. Exceptions to this rule may be due to the fact that nOH and nSH could manifest with different degrees of severity according to the time of the day, intravascular volume status and timing of the administration of anti-hypotensive drugs. Anti-hypotensive drugs may succeed in raising the standing BP and improving symptoms, but exacerbate nSH.

A diagnosis of nSH should be also made independently from seated BP values, which may vary from hypertensive values to normo- or hypotensive values in patients with cardiovascular autonomic failure.

In addition to office BP screening, home BP recordings performed by patients themselves are recommended to gather further insights into circadian BP control. Although no protocol for home BP self-monitoring has been validated in patients with cardiovascular autonomic failure, we recommend that the BP be recorded three times per day (early morning, after lunch, at bedtime) in the supine, seated and standing position for 1 week at first diagnostic work-up. The same home BP protocol should be repeated after the initiation or adaptation of any treatment for nOH or nSH in order to monitor therapeutic effects and to detect potential side effects. Comparison of supine and seated BP values during the daytime is particularly valuable for choosing therapeutic strategies: patients with nSH may show normal BP values while seated, hence benefitting from a simple non-pharmacological approach such as avoiding the supine position during daytime.

If office BP screening or home BP recordings suggest nSH, a 24 h-ABPM is recommended to ascertain the presence of nocturnal hypertension and document absolute BP levels reached overnight. 24 h-ABPM may also provide information on the severity of nOH during daily activity or after meals (“post-prandial hypotension” [56]), thus supporting customization of the therapeutic management [57].

Additional considerations are required when interpreting 24 h-ABPM data of patients with cardiovascular autonomic failure:

- Several activities of daily life may cause severe drops in BP, which may be captured in the 24-h recordings. For an accurate interpretation of the 24 h-ABPM data, patients should be instructed to compile an activity diary on the examination day in which they report the times of medication intake (especially anti-hypotensive and anti-hypertensive agents), meals, physical activities and times of getting out of bed at night (for example, to use the bathroom).
- Extreme drops in BP during daytime will reduce mean BP during the daytime, thereby resulting in an overestimation of the percentage of nocturnal BP rise. Conversely, nocturnal standing or bathroom visits may induce nocturnal BP falls, which may result in an underestimation of the BP rise overnight. For these reasons, both the percentage of nocturnal BP rise, as well as the absolute overnight BP values should be considered when diagnosing and tailoring treatment strategies for nSH. Whereas non-dipping and rising nocturnal patterns have been associated with long-term end-organ damage, absolute BP values reached while supine may be more relevant for the development of hypertensive emergencies.
- Sleeping with the bed 12° head-up tilted (or higher, if tolerated) creates an overnight gravitational stress and is an effective non-pharmacological strategy to manage nOH [58]. Sleeping with the whole bed tilted can mitigate the severity of nocturnal nSH by inducing venous pooling below the level of the heart throughout the night. Sleeping with only the head of the bed raised appears to be less effective in lowering night-time BP. Close attention should be therefore paid to the angle of the bed when interpreting the 24 h-ABPM data.
- Patients with cardiovascular autonomic failure, especially in the setting of PD and MSA, may suffer from sleep fragmentation or sleep disordered breathing, both of which have been associated with development of hypertension [59–61]. If sleep disordered breathing is suspected at clinical history taking or based on 24 h-ABPM data (for example, by documenting multiple nocturnal hypertensive peaks), a targeted diagnostic work-up is indicated.
- Nocturnal BP profiles may change over the short term with repeated 24 h-ABPM in 10–35% patients with essential hypertension [62, 63]. Reproducibility of the 24 h-ABPM readings in patients with cardiovascular autonomic failure remains to be investigated. This may be important as BP can fluctuate on a day-to-day basis due to multiple factors.

Finally, if a nocturnal BP rise is documented in a patient not known to suffer from any autonomic disorder, screening for nOH may be indicated. Similarly, if a patient is found to have elevated BP while supine—for example, in a hospital or acute care setting—they should also have the BP measured seated and, if the systolic difference exceeds 10 mmHg, standing as well. Otherwise, nSH might be mistaken for essential hypertension, and if treatment decisions are based on supine BP values alone, medications could potentially worsen unrecognized nOH.

## Perspectives

Current epidemiological data are not sufficient to define cut-offs for nSH based on its impact on end-organ damage or mortality. For this reason, the criteria proposed here for the diagnosis of nSH in the context of cardiovascular autonomic failure are based on the criteria for the diagnosis of essential hypertension and interpretation of 24 h-ABPM recordings in the general population [7, 8]. There are several reasons supporting this strategy: first, to facilitate the use of these cut-off values in clinical practice; second, to apply severity degrees of nSH as a tool for customizing treatment and grade adverse events in future interventional clinical trials for nOH; third, to generate data which can be compared across other epidemiological cohorts, such as essential hypertension. These definitions of nSH should ultimately lead to a better understanding of the prognostic outcome and stratification of the risk of adverse events.
